# Impairment of Neutrophil Migration to Remote Inflammatory Site during Lung Histoplasmosis

**DOI:** 10.1155/2015/409309

**Published:** 2015-01-29

**Authors:** Alexandra I. Medeiros, Adriana Secatto, Caroline Bélanger, Carlos A. Sorgi, Pierre Borgeat, Sylvie Marleau, Lúcia H. Faccioli

**Affiliations:** ^1^School of Pharmaceutical Sciences, UNESP, 14801-902 Araraquara, SP, Brazil; ^2^Faculdade de Ciências Farmacêuticas de Ribeirão Preto, Universidade de São Paulo, 14040-903 Ribeirão Preto, SP, Brazil; ^3^Faculty of Pharmacy, Université de Montréal, Montréal, QC, Canada H3T 1J4; ^4^Centre de Recherche en Rhumatologie et Immunologie, Centre de Recherche du CHUQ (CHUL), Sainte-Foy, QC, Canada G1V 4G2

## Abstract

*Histoplasma capsulatum* (*Hc*) induces a pulmonary disease in which leukotrienes promote activation and recruitment of effectors cells. It is also well-recognized that leukotriene B_4_ (LTB_4_) and platelet-activating factor (PAF) induce leukocyte recruitment to inflammatory sites. We investigated the impact of pulmonary *Hc* infection on PMN migration to a remote inflammatory site. Our results show that pulmonary *Hc* infection impairs LTB_4_- or PAF-stimulated PMN recruitment to air pouch. Yet, remote inflammation did not modify PMN numbers in the bronchoalveolar lavage fluid (BALF) of *Hc*-infected mice. Interestingly, the concomitant administration of PAF and LTB_4_ receptor antagonists inhibited PMN recruitment to both BALF and the remote site, demonstrating cooperation between both mediators. Along that line, our results show that PAF-elicited PMN chemotaxis was abrogated in 5-lipoxygenase-deficient animals. These results suggest caution in the indiscriminate use of anti-inflammatory drugs during infectious diseases.

## 1. Introduction

*Histoplasma capsulatum* (*Hc*), a major opportunistic dimorphic fungus, infects a growing number of immunocompromised individuals following the inadvertent inhalation of the spores or mycelial fragments [1, 2]. Hence, the most common manifestation of* Hc* infection is a pulmonary disease characterized by a chronic granulomatous and suppurative inflammatory reaction. Lipid mediators, such as leukotriene B_4_ (LTB_4_) and platelet-activating factor (PAF), are produced both by infiltrating inflammatory cells and airway epithelial cells and act in a cooperative manner to promote extravascular polymorphonuclear neutrophil (PMN) accumulation in response to various stimuli [3, 4]. In agreement, the levels of LTB_4_ are elevated in lungs of* Hc*-infected mice [5] and experimental evidence points to a cooperative role of LTB_4_ and PAF in inducing the formation of lipid bodies within leukocytes, thus amplifying eicosanoid generation in the course of fungal infection [6]. Although a marked neutrophil leukocytosis usually accompany the acute inflammatory response during the primary infection, whether these leukocytes are able to migrate to a remote secondary site of inflammation is not known. The aim of this study was to investigate the influence of acute* Hc* infection on PMN recruitment to remote localized inflammatory site induced by soluble LTB_4_ or PAF, and also to delineate the effect of LTB_4_- and/or PAF-receptor antagonists, on PMN numbers in the lungs of* Hc*-infected mice and at a remote location. Our results show that in* Hc*-infected mice PMN recruitment to remote inflammatory site is impaired, whereas the inoculation of LTB_4_ or PAF in air pouch did not affect lung inflammation induced by the infection.

## 2. Methods

### 2.1. Animals

Six- to eight-week-old C57BL/6 or 5-LO-deficient (5-LO^−/−^) (129-Alox5^tmI/Fun^) male mice were bred in the Faculdade de Ciências Farmacêuticas de Ribeirão Preto (Universidade de São Paulo, Ribeirão Preto, Brazil). They were maintained in a room at 25°C with a light/dark cycle of 12 h and free access to food and water. Infected animals were kept in biohazard facilities and housed in cages within a laminar flow safety enclosure under standard conditions. Experiments were approved by and conducted in accordance with guidelines of the Universidade de São Paulo Animal Care Committee.

### 2.2. Reagents

(+)-1-(3S,4R)-[3-(4-phenyl-benzyl)-4hydroxy-chroman-7-yl]-cyclopentan carboxylic acid (CP-105,696) was kindly provided by Dr. D. W. Owens (Pfizer, Groton, CT, USA) and [N-(2-dimethylamino-ethyl)-N-(3-pyridinylmethyl)[4-(2,4,6-triisopropyl-phenyl)thiazol-2-yl] amine] (SR-27417) by Dr. J.-M. Herbert (Sanofi-Aventis, Toulouse, France). PAF was purchased from Sigma-Aldrich (St-Louis, MO, USA) and LTB_4_ from Cayman Chemical Company (Ann Arbor, MI, USA), respectively.

### 2.3. Drug Administration

CP-105,696 and SR-27417 were dissolved in appropriate vehicles, 0.5% carboxymethylcellulose (CMC) and sterile water, respectively. CP-105,696 (1 mg/kg) and SR-27417 (0.1 mg/kg) were administered orally, 16 and 2 h before s.c. injections of agonists in air pouch, respectively. Vehicle-treated mice received an oral administration of CMC or water and were fasted by removing the solid pellets (but not water) 16 h prior to the experiment.

### 2.4. Preparation of* H. capsulatum* and Infection

The clinical isolate of* H. capsulatum* obtained from a patient at the Hospital das Clínicas da Faculdade de Medicina de Ribeirão Preto, USP, was grown and prepared as previously described [5]. Yeast cells were used when fluorescein diacetate and ethidium bromide staining revealed their viability to be ≥90%. C57Bl/6 mice were anesthetized i.p. with 2.5% 2, 2, 2-tribromoethanol and inoculated intratracheally (i.t.) with 5 × 10^5^ live yeast cells in 100 *μ*L PBS, as described [5]. 5-LO^−/−^ mice were also infected with 3 × 10^6^ live yeast cells in 100 *μ*L PBS [7].

### 2.5. Experimental Protocol

Air pouches were raised on the dorsum of mice by s.c. injection of 3 and 2.5 mL of sterile air on days 0 and 3, respectively. On day 4, mice were infected with* Hc*. On day 6, 1 mL of LTB_4_ (0.1 *μ*g/mL), PAF (1 *μ*g/mL), or PBS was injected into the air pouches. After 4 h, mice were put in a CO_2_ chamber and blood was collected by cardiac puncture. Cells from the bronchoalveolar space were collected as described before [5]. Air pouches were washed with cold PBS and the exudates were centrifuged at 300 ×g for 10 min at 8°C and the pellets resuspended in 1 mL of PBS. Total and differential cell counts were quantitated using a hemacytometer and Panoptic-stained blood smears and air pouch cytocentrifuged preparations.

### 2.6. Statistical Analysis

All results are expressed as mean ± SEM. One-way analysis of variance (ANOVA) with Student-Newman-Keuls multiple comparisons posttest was used to compare differences between groups using GraphPad Prism Version 4.0 (San Diego, CA). Differences were considered significant at *P* < 0.05.

## 3. Results

### 3.1. Primary Infection in the Lung Impairs PMN Recruitment to a Remote Inflammatory Site

**Figure 1:**
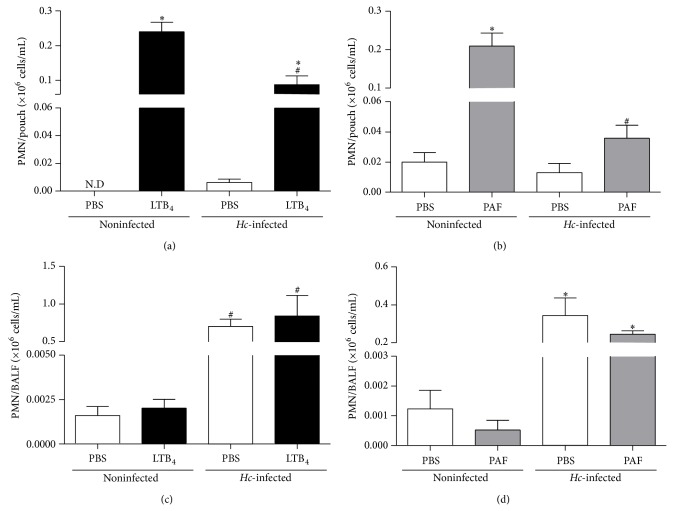
*Hc* lung infection impairs neutrophils recruitment to the remote air pouch. (a and c) LTB_4_ (0.1 *μ*g) or (b and d) PAF (1 *μ*g) were injected in 1 mL PBS, 2 days after* Hc* infection in C57Bl/6 mice (i.t., 5 × 10^5^ *Hc* yeast) (*Hc*-infected) or PBS (i.t., 100 *μ*L) (noninfected). PBS was injected in air pouch as control (*n* = 3). Four hours after agonist inoculation in air pouch mice were killed and the cells in (a, b) air pouch and in (c, d) BALF were obtained and PMN in both compartments were counted as described in Materials and Methods. Data are the mean ± SEM of *n* = 4–8 (a and b) or *n* = 4–6 (c and d) ^*^ *P* < 0.05 versus PBS; ^#^ *P* < 0.05 noninfected versus* Hc*-infected mice.

In a first series of study, we assessed PMN recruitment to a remote site of inflammation in mice harboring concomitant pulmonary* Hc* infection. As shown in Figures 1(a) and 1(b), administration of soluble agonist, such as LTB_4_ or PAF into the air pouch cavity, elicited an intense inflammatory cell infiltrate consisting primarily of PMN into the air pouch of noninfected mice when compared to vehicle inoculation. In contrast, LTB_4_- or PAF-elicited PMN recruitment to the air pouch was greatly hindered in* Hc*-infected mice versus uninfected mice, when comparing match given stimuli. As expected, intratracheal inoculation with* Hc* led to significant PMN accumulation into the BALF of* Hc*-infected mice (Figures 1(c) and 1(d)). Yet, whether or not LTB_4_ (Figure 1(c)) or PAF (Figure 1(d)) was injected locally into the air pouch cavity to induce an acute secondary inflammatory response, PMN were still efficiently recruited to the infected lungs (Figures 1(c) and 1(d)). These observations were not attributable to changes in circulating cell numbers inasmuch as no significant differences in PMN blood cells were found between groups 48 h after* Hc* inoculation (data not shown).

### 3.2. Effect of PAF- and LTB_4_-Antagonists on PMN Recruitment

**Figure 2:**
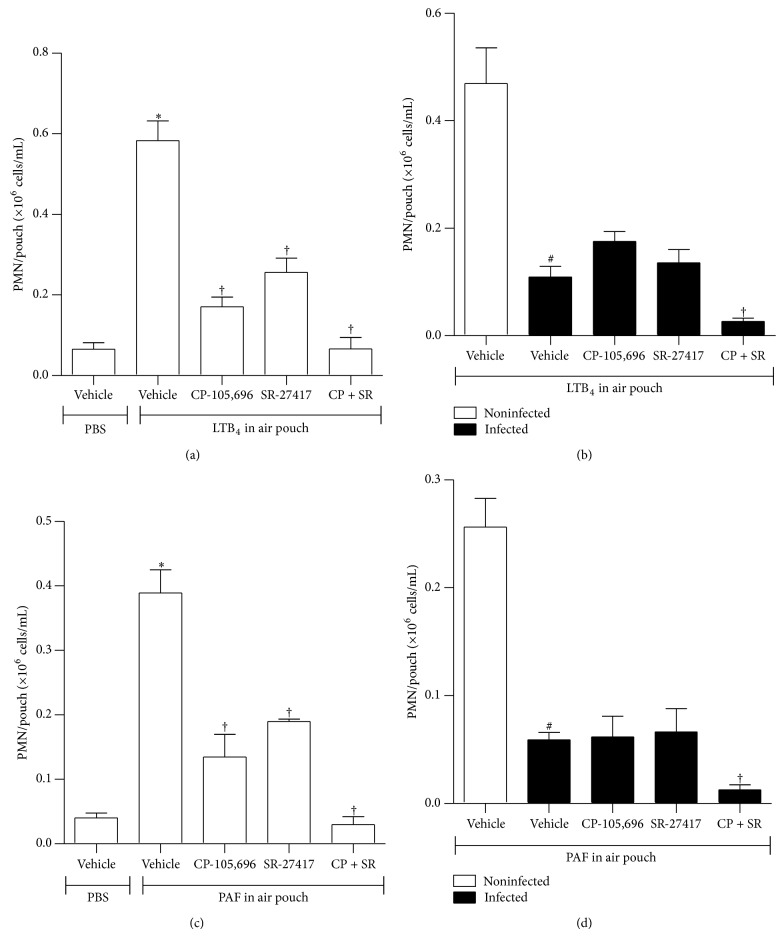
Effect of SR-27417 and CP-105,696 administration on PMN numbers in air pouch of C57Bl/6 mice. (a and c) Noninfected mice and (b and d)* Hc*-infected mice (i.t., 5 × 10^5^ *Hc* yeast) were pretreated with SR-27417 (0.1 mg/kg) and/or CP-105,696 (1 mg/kg) orally 2 and 16 h, respectively, before infection. (a and b) LTB_4_ (0.1 *μ*g) or (c and d) PAF (1 *μ*g) injection (in 1 mL PBS vehicle) into the air pouch. Vehicle-treated mice received an oral administration of 0.5% CMC or water. PMN numbers in air pouch were determined as described in Materials and Methods. Data are the mean ± SEM of *n* = 6–12 (a and b) or of *n* = 6–8 (c and d). ^*^ *P* < 0.05 versus vehicle noninfected mice (PBS into air pouch); ^#^ *P* < 0.05 versus noninfected mice (agonist into air pouch). ^†^ *P* < 0.05 versus infected mice (agonist into air pouch).

In another series of experiments, we investigated whether administration of specific anti-inflammatory drugs promotes further blockade of PMN influx to the air pouch cavity of infected mice. To this aim, mice were pretreated orally with either CP-105,696, a selective and potent noncompetitive antagonist of BLT1 receptors [8] or SR-27417, a selective and potent competitive PAF receptor antagonist [9], prior to agonist inoculation but after* Hc* infection. In other work, we demonstrated the efficacy for the CP-105,696 and SR-27417 treatment in the inhibition of LTB_4_- and PAF-elicited PMN accumulation in the dermis [3]. In our experiments, CP-105,696 (1 mg/kg) inhibited LTB_4_-elicited PMN accumulation by 71% (*P* < 0.05) (Figure 2(a)) and PAF-elicited PMN accumulation by 65% (*P* < 0.05) (Figure 2(c)) in the air pouch. CP-105,696 or SR-27417 pretreatment also partially inhibited PMN recruitment elicited by PAF or LTB_4_. The concomitant administration of CP-105,696 and of SR-27417 exerted a cooperative inhibitory effect on PMN recruitment by either agonist (Figures 2(a) and 2(c)). In contrast, a single administration of CP-105,696 did not further decrease LTB_4_-elicited PMN influx to the air pouch cavity 48 h after inoculating* Hc* in C57Bl/6 mice (Figure 2(b)). Similar observations were made following pretreatment with a single oral dose of SR-27417: the reduced PMN infiltration in response to locally injected PAF in air pouch was not further inhibited by the antagonist (Figure 2(d)). Yet, a concomitant pretreatment with CP-105,696 and SR-27417 of infected animal was associated with a profound (~95%) inhibition of PMN accumulation stimulated by either agonists (Figures 2(b) and 2(d)).

**Figure 3:**
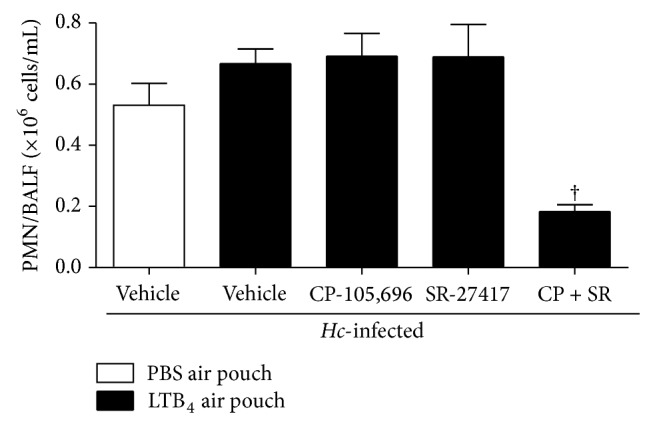
Effect of SR-27417 and CP-105,696 on PMN numbers in BALF of* Hc*-infected mice.* Hc*-infected mice (i.t., 5 × 10^5^ *Hc* yeast) were pretreated with SR-27417 (0.1 mg/kg) and/or CP-105,696 (1 mg/kg) orally 2 and 16 hours, respectively, before LTB_4_ (0.1 *μ*g) injection (in 1 mL PBS) into the air pouch. Vehicle-treated mice received an oral administration of 0.5% CMC or water. BALF was obtained 2 days after* Hc* infection and PMN numbers determined as described in Materials and Methods. Data are the mean ± SEM of *n* = 6–12. ^†^ *P* < 0.05 versus vehicle (LTB_4_ into air pouch).

We next investigated whether a systemic treatment with the anti-inflammatory drugs, CP-105,696 and SR-27417, would interfere with PMN recruitment in the BALF of* Hc*-infected mice. Our results show that only a concomitant administration of CP-105,696 and SR-27417 significantly decreased PMN numbers by 72% (*P* < 0.05) in the BALF of* Hc*-infected mice (Figure 3).

### 3.3. Role of Endogenous Leukotrienes in PAF-Elicited PMN Recruitment

**Figure 4:**
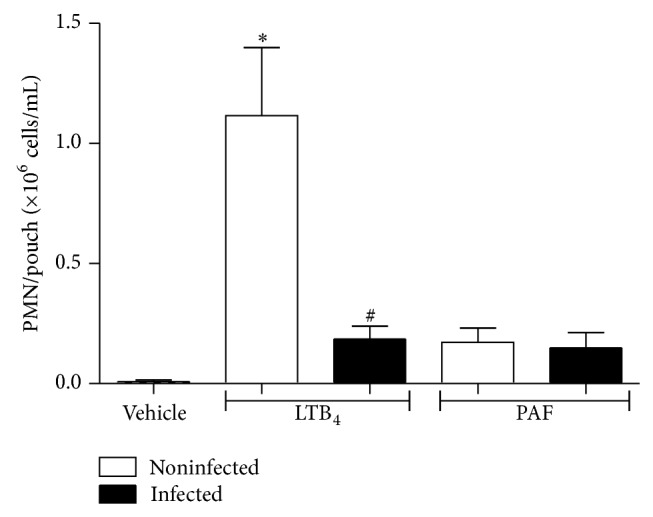
PMN recruitment to air pouch induced by PAF is dependent on endogenous leukotrienes. 5-LO^−/−^ mice were inoculated with PBS or infected with* Hc* (i.t., 3 × 10^6^ yeast). After 2 days, LTB_4_ (0.1 *μ*g), PAF (1 *μ*g), or PBS (1 mL) were injected into air pouch, and, 4 h after, PMN were recovered and the counts were determined as described in Materials and Methods. Data are the mean ± SEM of *n* = 4-5. ^*^ *P* < 0.05 versus vehicle (noninfected mice); ^#^ *P* < 0.05 versus noninfected (LTB_4_ into air pouch).

The enhanced inhibitory effect of the concomitant administration of CP-105,696 and SR-27417 on soluble agonists-induced PMN recruitment to the remote, localized inflammatory site in both uninfected and* Hc*-infected mice, suggest a cross-talk between PAF and elicited-endogenous leukotrienes. To corroborate this hypothesis, 5-LO^−/−^ mice were infected (or not) with* Hc* and air pouches were stimulated with either LTB_4_ or PAF for 4 h. As shown in Figure 4, exogenous LTB_4_ induced significant PMN recruitment to the air pouch of uninfected mice. In contrast,* Hc* infection in 5-LO^−/−^ mice, devoid of endogenous leukotrienes, impaired PMN migration to the air pouch induced by LTB_4_. However, deficiency of endogenous leukotrienes impaired PMN recruitment into the air pouch induced by PAF in both noninfected and* Hc*-infected mice, supporting that PAF is critically dependent upon the activation of 5-LO for optimal chemotactic response.

## 4. Discussion

A major finding of the present studies is that, in a murine model with an inflammatory complication remote from the* Hc*-infected lung, PMN emigration to lungs is preserved; however, the local cell migration at the distant inflammatory site is impaired. In the present study, mice were inoculated intratracheally with* Hc* yeast cells, to more closely mimic the natural way of acquiring the infection by the inhalational route in humans [10]. Acute pulmonary histoplasmosis is associated with a strong PMN predominance in lungs and BALF fluid, where these cells exhibit fungistatic activity against* Hc* in both human [11–13] and mice [5, 14]. We demonstrated previously at day 2 after* Hc *infection that there were the production of leukotrienes (LTB_4_ and LTC_4_) as well as high levels of KC (murine IL-8 homologue) and TNF-*α* [5]. The presence of these mediators in the lung during the* Hc *infection could explain the high number of PMN to the lung when compared to peripheral inflammation. Moreover, the lung infiltrating leukocytes presented a higher number of lipid bodies [6], which are an important source of inflammatory lipid mediators in various inflammatory and/or infectious contexts [4, 15, 16], along with PAF [17, 18]. In agreement, it was shown that both LTB_4_ and PAF exerted autocrine effects on leukotriene synthesis through an increase in arachidonic acid bioavailability [20] thereby emphasizing the interplay between LTB_4_ and PAF and their cooperative actions on PMN recruitment at inflammatory sites [3]. The observations further supported the investigation of these mediators as soluble agonists of the remote inflammatory response in the air pouch of* Hc*-infected mice. The availability of potent and selective LTB_4_ and PAF receptor antagonists, and of 5-LO-deficient mice, allowed assessment of the potential of these anti-inflammatory drugs to further inhibit PMN recruitment at both primary and remote inflammatory sites. Our results show that only the concomitant pharmacological blockade of LTB_4_ and PAF receptors exerts an inhibitory effect on either LTB_4_- or PAF-induced PMN migration, both at the site of infection and at a site remote to the original insult. These results are in agreement with our previous observations that PAF and LTB_4_ exert important cooperative effect at the primary site of infection [6]. Our results also support a cooperative effect of these mediators on PMN recruitment at the remote inflammatory site. Whereas administration of a single antagonist partially blocked the recruitment of PMN in the pouch in uninfected mice, the concomitant administration of the drug antagonists CP-105,696 and SR-27417 blocked PMN influx by more than 90%. Recruitment at a remote location was greatly blunted. Furthermore the administration of a single dose of either PAF or LTB_4_ antagonists did not further decrease pouch PMN recruitment, whereas the concomitant administrations of the drugs (p.o.) further decreased PMN recruitment in the pouch of infected mice. In contrast, the concomitant administrations of the drugs further decrease PMN recruitment into the pouch of infected mice. These results further support reliance on endogenous lipid mediators biosynthesis for the effective recruitment of PMN in response to exogenous LTB_4_ and PAF administration. This hypothesis was confirmed when we evaluated the effect of PAF agonist into the air pouch of 5-LO^−/−^ mice. The 5-LO deficiency impaired the PMN recruitment into air pouch in response to PAF demonstrating the cross talking between endogenous LTs and PAF in secondary inflammatory site occurring concomitantly with a primary vigorous primary infection. They may also suggest that PMN recruitment at secondary inflammatory sites may be further decreased by drugs which may block lipid mediator biosynthesis, in addition to the inhibitory effect of primary infection itself.

## 5. Conclusions

In summary, our results show that after 2 days of infection with* Hc*, PMN emigration is decreased in both LTB_4_- and PAF-inoculated dorsal air pouch, used as a model of remote inflammation. In contrast, cell numbers in infectious lungs, as a primary site, are preserved whether the animals received LTB_4_ or PAF into the air pouch. These results support that PMN recruitment is dependent on redundant release of mediators into* Hc*-infected lungs in addition to lipid mediators including, among others, the cytokines TNF-*α*, IL-1, IL-12, and IFN-*γ* that were shown to play an important role in the control of the disease and may contribute to PMN recruitment and the control of pathogen replication. Our results may also suggest use with caution of anti-inflammatory agents that may prevent the endogenous release of lipid mediators in order to avoid PMN decrease at the site of infection.
